# Notification that new names of prokaryotes, new combinations, and new taxonomic opinions have appeared in volume 74, part 4 of the IJSEM

**DOI:** 10.1099/ijsem.0.006391

**Published:** 2024-07-31

**Authors:** Aharon Oren, Markus Göker

**Affiliations:** 1 The Institute of Life Sciences, The Hebrew University of Jerusalem, The Edmond J. Safra Campus, 9190401 Jerusalem, Israel; 2 Leibniz Institute DSMZ – German Collection of Microorganisms and Cell Cultures, Inhoffenstrasse 7B, 38124 Braunschweig, Germany

This listing of names of prokaryotes published in a previous issue of the *International Journal of Systematic and Evolutionary Microbiology* is provided as a service to microbiology to assist in the recognition of new names and new combinations. This procedure was proposed by the Judicial Commission [1]. The names given herein are listed according to the Rules of priority (i.e., time and date of publication of the articles on the journal’s platform and the order of valid publication of names in the original articles) [2].

**Table:** 

Order of article publication	Name/authors	Proposed as	Article no.	Page no.
1	*Flavobacterium psychraquaticum* Avendaño-Herrera *et al*. 2024	sp. nov.	006309	9
2	*Actomonas* Zhang *et al*. 2024	gen. nov.	006314	7
2	*Actomonas aquatica* Zhang *et al*. 2024	sp. nov.	006314	7
3	*Blastococcus brunescens* Hezbri *et al*. 2024	sp. nov.	006317	7
4	*Marinobacter azerbaijanicus* Sánchez-Porro *et al*. 2024	sp. nov.	006308	10
5	*Ruegeria marisflavi* He *et al*. 2024	sp. nov.	006323	6
5	*Ruegeria aquimaris* He *et al*. 2024	sp. nov.	006323	7
6	*Rubellicoccus* Luo *et al*. 2024	gen. nov.	006325	4
6	*Rubellicoccus peritrichatus* Luo *et al*. 2024	sp. nov.	006325	5
7	*Flagellimonas baculiformis* Wang *et al*. 2024	sp. nov.	006316	8
7	*Flagellimonas crocea* Wang *et al*. 2024	sp. nov.	006316	8
8	*Shuttleworthella* Deshmukh and Oren 2024	gen. nov.	006318	1
8	*Shuttleworthella satelles* (Downes *et al*. 2002) Deshmukh and Oren 2024	comb. nov. [basonym: *Shuttleworthia satelles* Downes *et al*. 2002]*	006318	2
8	*Nostocoides* Deshmukh and Oren 2024	gen. nov.	006318	2
8	*Nostocoides australiense* (Maszenan *et al*. 2000) Deshmukh and Oren 2024†	comb. nov. [basonym: *Tetrasphaera australiensis* Maszenan *et al*. 2000]	006318	2
8	*Nostocoides japonicum* (Maszenan *et al*. 2000) Deshmukh and Oren 2024	comb. nov. [basonym: *Tetrasphaera japonica* Maszenan *et al*. 2000]	006318	2
8	*Nostocoides jenkinsii* (McKenzie *et al*. 2006) Deshmukh and Oren 2024	comb. nov. [basonym: *Tetrasphaera jenkinsii* McKenzie *et al*. 2006]§	006318	3
8	*Nostocoides vanveenii* (McKenzie *et al*. 2006) Deshmukh and Oren 2024	comb. nov. [basonym: *Tetrasphaera vanveenii* McKenzie *et al*. 2006]	006318	3
8	*Nostocoides veronense* (McKenzie *et al*. 2006) Deshmukh and Oren 2024	comb. nov. [basonym: *Tetrasphaera veronensis* McKenzie *et al*. 2006]	006318	3
9	*Isoptericola luteus* Bing *et al*. 2024	sp. nov.	006324	7
10	*Aneurinibacillus uraniidurans* Zhang *et al*. 2024	sp. nov.	006297	6
11	*Brooklawnia propionicigenes* Akita *et al*. 2024	sp. nov.	006320	9
12	*Sphingobacterium allocomposti* Goo and Li 2024	nom. nov. [basonym: *Sphingobacterium composti* Yoo *et al*. 2007]; illegitimate homotypic synonym: *Sphingobacterium composti* Yoo *et al*. 2007	006319	2
12	*Mycobacterium chelonae* subsp. *bovistauri* Goo and Li 2024	nom. nov. [basonym: *Mycobacterium chelonae* subsp. *bovis* Kim *et al*. 2017]; illegitimate homotypic synonym: *Mycobacterium chelonae* subsp. *bovis* Kim *et al*. 2017*	006319	2
12	*Lactobacillus delbrueckii* subsp. *allosunkii* Goo and Li 2024	nom. nov. [basonym: *Lactobacillus delbrueckii* subsp. *sunkii* Kudo *et al*. 2012]; illegitimate homotypic synonym: *Lactobacillus delbrueckii* subsp. *sunkii* Kudo *et al*. 2012	006319	3
12	*Christiangramia oceanisediminis* (Yang *et al*. 2023) Goo and Li 2024	comb. nov. [basonym: *Gramella oceanisediminis* Yang *et al*. 2023]; illegitimate homotypic synonym: *Gramella oceanisediminis* Yang et al. 2023	006319	3
12	*Christiangramia crocea* (Zhang *et al*. 2023) Goo and Li 2024	comb. nov. [basonym: *Gramella crocea* Zhang *et al*. 2023]; illegitimate homotypic synonym: *Gramella crocea* Zhang *et al*. 2023	006319	4
13	*Pseudomonas kulmbachensis* Lick *et al*. 2024	sp. nov.	006293	7
13	*Pseudomonas paraveronii* Lick *et al*. 2024	sp. nov.	006293	14
14	*Marinobacter qingdaonensis* Shao *et al*. 2024	sp. nov.	006327	8
15	*Chryseobacterium metallicongregator* Spotts *et al*. 2024	sp. nov.	006337	6
16	*Micromonospora cathayae* Long *et al*. 2024	sp. nov.	006332	7
17	*Kaistella rhinocerotis* Park *et al*. 2024	sp. nov.	006338	6
17	*Kaistella faecalis* (Son *et al*. 2022) Park *et al*. 2024	comb. nov. [basonym: *Chryseobacterium faecale* Son *et al*. 2022]	006338	8
18	*Methylomonas defluvii* Ouyang *et al*. 2024	sp. nov.	006321	9
19	*Paenibacillus thermotolerans* Xiang *et al*. 2024	sp. nov.	006336	9
20	*Chelativorans salis* Gao *et al*. 2024	sp. nov.	006340	7
21	*Methylopilaceae* diCenzo *et al.* 2024	fam. nov.	006328	11
21	*Rhodoblastaceae* diCenzo *et al.* 2024	fam. nov.	006328	11
21	*Rhodoligotrophaceae* diCenzo *et al.* 2024	fam. nov.	006328	11
21	*Salaquimonadaceae* diCenzo *et al.* 2024	fam. nov.	006328	11
22	*Achromobacter seleniivolatilans* Zhang *et al.* 2024	sp. nov.	006334	8
22	*Buttiauxella selenatireducens* Zhang *et al.* 2024	sp. nov.	006334	8
23	*Massilia luteola* Huang *et al.* 2024	sp. nov.	006331	7
24	*Microbaculum marinisediminis* Zhang *et al.* 2024	sp. nov.	006311	5
25	*Pectobacterium araliae* Sawada *et al.* 2024	sp. nov.	006326	9
26	*Chryseotaleaceae* Lu *et al.* 2024	fam. nov.	006345	8
26	*Flectobacillaceae* Lu *et al.* 2024	fam. nov.	006345	9
26	*Leadbetterellaceae* Lu *et al.* 2024	fam. nov.	006345	9
26	*Arcicella gelida* Lu *et al.* 2024	sp. nov.	006345	9
26	*Arcicella lustrica* Lu *et al.* 2024	sp. nov.	006345	10
27	*Sphingobium cyanobacteriorum* Le *et al.* 2024	sp. nov.	006339	8
28	*Lentzea sokolovensis* Lara *et al.* 2024	sp. nov.	006335	9
28	*Lentzea kristufekii* Lara *et al.* 2024	sp. nov.	006335	10
28	*Lentzea miocenica* Lara *et al.* 2024	sp. nov.	006335	10
29	*Peiella* Wu *et al.* 2024	gen. nov.	006344	7
29	*Peiella sedimenti* Wu *et al.* 2024	sp. nov.	006344	7
30	*Falsiroseomonas oryziterrae* Lee and Whang 2024	sp. nov.	006349	7
30	*Falsiroseomonas oryzae* Lee and Whang 2024	sp. nov.	006349	7
31	*Methanooceanicella* Zhang *et al.* 2024	gen. nov.	006322	8
31	*Methanooceanicella nereidis* Zhang *et al.* 2024	sp. nov.	006322	8
32	*Streptomyces profundus* Ribeiro *et al.* 2024	sp. nov.	006341	7
33	*Terrirubrum* Guo *et al.* 2024	gen. nov.	006348	9
33	*Terrirubrum flagellatum* Guo *et al.* 2024	sp. nov.	006348	9
33	*Terrirubraceae* Guo *et al.* 2024	fam. nov.	006348	10
33	*Lichenibacterium dinghuense* Guo *et al.* 2024	sp. nov.	006348	10
33	*Rhodoblastaceae* Guo *et al.* 2024‡	fam. nov.	006348	10
34	*Apirhabdus* Quinn *et al.* 2024	gen. nov.	006346	10
34	*Apirhabdus apintestini* Quinn *et al.* 2024	sp. nov.	006346	10
35	*Paucibacter sediminis* Jung *et al.* 2024	sp. nov.	006312	7
36	*Bifidobacterium apis* Jiang *et al.* 2024	sp. nov.	006358	4
37	*Paraburkholderia translucens* Oh *et al.* 2024	sp. nov.	006347	7
37	*Paraburkholderia sejongensis* Oh *et al.* 2024	sp. nov.	006347	8
38	*Ruixingdingia* Xu *et al.* 2024	gen. nov.	006350	7
38	*Ruixingdingia sedimenti* Xu *et al.* 2024	sp. nov.	006350	8
39	*Psychrobacter raelei* Manzulli *et al.* 2024	sp. nov.	006353	8
40	*Aquincola agrisoli* Huq *et al.* 2024	sp. nov.	006355	8
41	*Anaerolentibacter* Yan *et al.* 2024	gen. nov.	006359	9
41	*Anaerolentibacter hominis* Yan *et al.* 2024	sp. nov.	006359	9
41	*Diplocloster hominis* Yan *et al.* 2024	sp. nov.	006359	9
41	*Pilosibacter* Yan *et al.* 2024	gen. nov.	006359	10
41	*Pilosibacter fragilis* Yan *et al.* 2024	sp. nov.	006359	10

*The basonym was assigned to Risk Group 2 (German TRBA 466, “Einstufung von Prokaryonten (Bacteria und Archaea) in Risikogruppen”).

†The etymology line should be corrected to *Nostocoides australiense* instead of *Allotetrasphaera australiense*.

§The etymology line should be corrected to *Tetrasphaera jenkinsii* McKenzie *et al.* 2006 instead of *Tetrasphaera jenkinsii* McKenzie *et al.* 2006 emend. Nouioui *et al.* 2018.

‡Illegitimate name; *Rhodoblastaceae* diCenzo *et al.* 2024 has priority.

The following taxonomic opinions (i.e., the emendation of circumscriptions or the creation of synonyms) do not affect the status of a name under the International Code of Nomenclature of Prokaryotes. These taxonomic opinions can neither be considered as approved nor as disapproved by the International Committee on Systematics of Prokaryotes.

**Table:** 

Name/authors:	Article no.	Page no.
*Bartonellaceae* Gieszczykiewicz 1939 (Approved Lists 1980) emend. diCenzo *et al.* 2024	006328	11
*Salipiger manganoxidans* (Rajasabapathy *et al.* 2016) Wirth and Whitman 2018 pro synon. *Salipiger marinus* (Lai *et al.* 2011) Wirth and Whitman 2018	006329	4
